# Clinical significance and efficacy of radiofrequency catheter ablation in the treatment of tachycardia-induced cardiomyopathy in 12 children

**DOI:** 10.1186/s13019-025-03830-y

**Published:** 2026-01-02

**Authors:** Min Zhang, Xiaoxiao Cao, Yong Zhang

**Affiliations:** The Children’s Heart Center, Tongji Medical College, Wuhan Children’s Hospital (Wuhan Maternal and child Healthcare Hospital, Huazhong University of Science & Technology100, 100 Hongkong Road, Jiangan District, Wuhan, Hubei China

**Keywords:** Tachycardia-induced cardiomyopathy, Children, Radiofrequency catheter ablation, Clinical characteristics

## Abstract

**Background:**

Tachycardia-induced cardiomyopathy (TIC) in children is significantly affected by factors such as type of arrhythmia and duration of the arrhythmia. Early detection and effective radiofrequency catheter ablation (RFCA) are highly beneficial in improving the prognosis of children with TIC. However, related research in this area is still limited, in particular with regard to detailed clinical characteristics and treatment efficacy in a specific pediatric population.

**Methods:**

A retrospective analysis was carried out on the medical records of TIC children admitted to Wuhan Children’s Hospital between January 2017 and March 2025. Clinical data, electrocardiograms, echocardiograms, treatment and follow-up methods were collected and statistically analysed. All children were subjected to intracardiac electrophysiological studies and a radiofrequency catheter ablation procedure after obtaining parental consent.

**Results:**

A total of 12 children were enrolled, distributed evenly between the sexes. The onset period of unemployment varied considerably. Arrhythmias included 7 cases of atrial tachycardia, 2 cases of atrioventricular reentrant tachycardia and 3 cases of ventricular tachycardia. Six children received ineffective antiarrhythmic drugs and finally received a RFCA. The left ventricular ejection fraction (LVEF) of children showed a remarkable increase after operation. Statistical analysis revealed a very significant difference in LVEF at 1 month post-RFCA compared to pre-RFCA (*P* = 0.002). At 1 month post-treatment, 75% of the children had LVEF above 50% and approximately 66.7% had LVEF above 55%. Improvements were maintained over time: six months after surgery 91.7% of children achieved a LVEF greater than 55% and a year after surgery all children achieved a LVEF of at least 100%. No recurrence of tachycardia was observed during the follow-up period.

**Conclusions:**

The most common cause of TIC in children is supraventricular tachycardia, particularly atrial tachycardia. RFCA is an effective treatment for pediatric TIC, allowing rapid recovery of left ventricular function. Clinicians should pay greater attention to TIC in children, use comprehensive screening methods for early diagnosis and provide early treatment to improve the prognosis of children with TIC.

**Supplementary Information:**

The online version contains supplementary material available at 10.1186/s13019-025-03830-y.

Tachycardia-induced cardiomyopathy (TIC), also called arrhythmia-induced cardiomyopathy (AiCM) [1], is influenced by three main factors: the type of arrhythmia, the duration of the arrhythmia and the level of the heart rhythm [1–3]. Types of tachycardia caused by cardiomyopathy include supraventricular tachycardia (including atrial tachycardia, permanent junctional tachycardia, and atrioventricular reentrant tachycardia) and ventricular tachycardia [4]. Atrial fibrillation is one of the most common causes of tachycardia-induced cardiomyopathy in adults [1, 3, 5]. In contrast, the main causes of TIC in children are atrial tachycardia and persistent atrioventricular junction tachycardia [2, 3, 5]. Clinically, TIC is typically manifested by findings such as cardiac enlargement and heart failure, both of which are driven by sustained rapid heart rates [1, 6, 7]. Some children are even at risk of sudden heart failure [1, 7]. Given the specific physiological characteristics of children, early detection, diagnosis and treatment are important to improve the prognosis for these children. Therefore, we summarized and analyzed the clinical characteristics of children with TIC admitted to Wuhan Children’s Hospital over the past seven years, as well as the characteristics and effectiveness of RFCA in treating this condition.

## Subjects and methods

### study subjects

This study comprehensively analyzed the medical records of children with TIC admitted to the Cardiovascular Ward of Wuhan Children’s Hospital between January 2017 and March 2025. The inclusion of study subjects was strictly limited to the following criteria [3]:

I. Multiple types of rapid arrhythmias have been reported, including atrial tachycardia, permanent junctional reciprocating tachycardia, various tachycardias caused by reentry mechanisms (such as atrioventricular reciprocating tachycardia, atrioventricular nodal reentrant tachycardia, etc.), frequent premature ventricular contractions, and ventricular tachycardia.
II. Clinical manifestations were characterised by tachypnea and pallor, and may be accompanied by additional symptoms including palpitations, chest pain, and reduced exercise tolerance.
III. Echocardiography showed an enlarged heart and a LVEF below 50%.
IV. Following antiarrhythmic therapy, tachycardia was effectively controlled, clinical signs significantly reduced, heart size returned to normal, and LVEF was at or above 55%.

#### Exclusion criteria

Patients with atrioventricular reentrant tachycardia (AVRT) or idiopathic left ventricular reentrant tachycardia (ILVT) who demonstrateLVEF improvement solely following tachycardia termination (without RFCA) were excluded. Such cases reflect transient hemodynamic impairment rather than established TIC.

### Research methods

In this study, a retrospective approach was used to collect relevant data from all children. Specifically, data included clinical characteristics (including age, gender and body weight), clinical manifestations, findings from physical examinations, electrocardiogram (ECG), 24 h holter electrocardiogram, echocardiography, treatment methods, detailed information on RFCA, key elctrophysiological findings, disease prognosis and follow-up at 1 month, six months and one year post-discharge.

During the study, all children were subjected to intracardiac electrophysiological studies performed by St. Jude’s Hospital, St. Paul, MN, USA, following informed consent and parental consent. All RFCA procedures adhered to the “green electrophysiology” protocol, with the primary goal of minimizing ionizing radiation exposure to ensure radiation safety in this pediatric cohort. During the RFCA, all children underwent electrophysiological studies and a revision of their blood test under general anesthesia. Electrophysiological catheters were inserted via the right femoral vein and left femoral vein, respectively. For activation mapping in ventricular tachycardia (VT), the EnSite system guided the placement of multi-electrode catheters at the right ventricular apex (RVA), coronary sinus (CS), and left ventricular septum to acquire relevant electrocardiographic data. In cases requiring evaluation of supraventricular tachycardia (SVT), selective mapping of the His bundle and right atrial appendage was performed concurrently to comprehensively assess cardiac electrophysiological characteristics.

### Statistical analysis

Statistical analyses for this study were performed using the SPSS 22.0 software package. The casualty number was expressed in percentages. Due to the small size of the sample and the non-normal distribution of the data set in this study, a Wilcoxon rank sum test was used to compare data prior to and following RFCA.

## Results

### General information

This study included a total of 12 children with TIC. Among them, there were 6 boys and 6 girls, reflecting an equal gender distribution (1:1). The age of initial onset of children spanned a wide range. The median age was 9 years and 6 months, with a range of 2 years and 11 months to 13 years.

Arrhythmia duration exhibited marked interpatient variability: patient 4 was admitted following 3 days of clinically documented tachycardia, with subsequent diagnosis of TIC. Given the well-recognized risk of delayed symptom recognition in pediatric patients—often due to variable ability to articulate cardiac symptoms—the actual arrhythmia onset in this case is likely longer than the 3-day documented period. The longest duration of intermittent tachycardia episodes across all patients was 6 years 4 months.

Regarding clinical manifestations, six children were primarily referred due to palpitations. One child presented with prominent pallor, another with persistent epigastric pain, and one with a syncopal episode. Notably, three children were asymptomatic, and their condition was incidentally identified during a routine physical examination (Table 1).

**Table 1:** Baseline characteristics and Post-RFCA therapeutic outcomes in pediatric patients with TIC

Patient	1	2	3	4	5	6	7	8	9	10	11	12
Gender	Boy	Boy	Girl	Girl	Girl	Girl	Boy	Boy	Girl	Girl	Boy	Boy
Age (years)	2.11	7.0	6.4	10.4	11.8	11.7	8.9	12.5	11.2	3.6	5.9	13.0
Weight (Kg)	15	19	23	48	36	54	28	92	38	15	23	34
Clinical Manifestation	Asymptomatic	Asymptomatic	Prominent pallor	Palpitation	Palpitation	Epigastric pain	Palpitation	Asymptomatic	Palpitation	Palpitation	Syncope	Palpitation
AADs	Digoxin + Amiodarone	None	Metoprolol + Amiodarone	None	None	Digoxin + ACEIs	Propafenone	None	None	None	IV Deslanoside	Amiodarone + Metoprolol
Diagnosis	VT	PVCs	VT	AVRT	AVRT	AT	AT	AT	AT	AT	AT	AT
Preop Max HR (bpm)	151	154	200	215	200	144	216	171	240	202	160	200
Localization of Arrhythmia	LPF	LAF	LAF	LAP	LAP	Right pulmonary vein	Right pulmonary vein	Left pulmonary vein	Left pulmonary vein	Right atrial appendage	Left atrial appendage	Right atrial appendage
Preop LVEDd (mm)	37	35	49	43	30	56	40	55	36	43	46	45
1-Month Postop LVEDd (mm)	35	37	/	44	42	57	39	53	39	37	37	43
6-Month LVEDD (mm)	36	38	46	44	41	48	38	53	38	35	31	40
Preoperative LVEF (%)	43	45	24	46	41	20	40	48	47	42	28	45
1-Month Postop LVEF (%)	*51*	*65*	*/*	*64*	*68*	*39*	*56*	*63*	*64*	*57*	*46*	*52*
6-Month Postop LVEF (%)	*57*	*64*	*42*	*60*	*67*	*57*	*63*	*64*	*64*	*57*	*58*	*57*
Hs-cTnT (ng/ml)	0.005	/	/	0.076	0.051	0.044	0.005	0.005	0.005	0.068	0.013	0.066
CK-MB (U/L)	48	23	22	16	18	21	25	13	19	14	17	17
NT-proBNP (pg/mL)	354	122	224	5419	2303	8066	429	80	136	9000	2768	181
Procedure Time (h: min)	2:18	2:00	2:00	1:41	1:38	3:00	2:10	2:20	2:00	2:00	3:00+ 1:10	1:40 + 1:00
Recurrent Tachycardia	No	No	No	No	No	No	No	No	No	No	No	No
Reablation	No	No	No	No	No	No	No	No	No	No	No	No
Recurrent TIC	No	No	No	No	No	No	No	No	No	No	No	No

Preop = Preoperative, Postop = Postoperative, Max = Maximum; Vent = Ventricular, bpm = beats per minute, LAF = Left Anterior Fascicle, LPF = Left Posterior Fascicle, PV = Pulmonary Vein, RAA = Right Atrial Appendage, LAA = Left Atrial Appendage, CS = Coronary Sinus, RVA = Right Ventricular Apex, LAP = Left Accessory Pathway, IV = Intravenous, Hs-cTnT = hypersensitive cardiac troponin, CK-MB = Creatine Kinase myocardial band, NT-proBNP = N-terminal pro-B type natriuretic peptide

**Table 2:** Electrophysiological features of pediatric patients

Patient	Diagnosis	Localization of arrhythmia	Key electrophysiological findings
1	VT	Left Posterior Fascicle (LPF)	ECG: LPF-origin VT; Earliest activation: mid-distal 1/3 of LPF; Ablation: VT terminated, no recurrence (incl isoproterenol [ISO])
2	PVCs	Left Anterior Fascicle (LAF)	ECG: LAF-origin PVCs; Earliest activation: proximal LAF; Ablation: PVCs eliminated, no recurrence (± ISO)
3	VT	Left Anterior Fascicle (LAF)	Baseline: sustained VT (atrioventricular [AV] dissociation); Earliest activation: proximal LAF (near left bundle branch trunk); Ablation: avoided (risk of complete left bundle branch block [cLBBB]), VT not terminated
4	AVRT	Left Accessory Pathway (LAP)	AVRT induced by ventricular pacing; Coronary sinus (CS) activation: centrifugal (earliest CS3/4); Optimal ventricular-atrial (VA) fusion: CS3/4-CS5/6; Ablation: VA dissociation, no recurrence
5	AVRT	Left Accessory Pathway (LAP)	AVRT induced by right ventricular apex (RVA) pacing; VA fusion: CS1/2; Ablation: VA dissociation (CS1/2), no recurrence (± ISO)
6	AT	Right Pulmonary Vein	Sustained AT (cycle length [CL] 430 ms); Right atrial (RA) mapping: unsuccessful; Earliest activation: left superior and inferior pulmonary veins (PVs); Ablation: AT terminated
7	AT	Right Pulmonary Vein	AT induced by ISO + high RA pacing; Earliest activation: inferior margin of right superior PV; Ablation: AT terminated
8	AT	Left Pulmonary Vein	Baseline: sustained AT; RA mapping: unsuccessful; Earliest activation: left inferior PV; Ablation: AT terminated
9	AT	Left Pulmonary Vein	Baseline: sustained AT (initial earliest activation CS1/2); Earliest activation: left superior and inferior PVs; Ablation: AT terminated
10	AT	Right Atrial Appendage	Baseline: sustained AT; Earliest activation: left PV region; Ablation: AT terminated
11	AT	Left Atrial Appendage	Baseline: sustained AT; Earliest activation: apex of left atrial appendage; Ablation: AT transiently terminated, recurrence (repeated stimulation)
12	AT	Right Atrial Appendage	Baseline: sustained AT; Earliest activation: mid-apical right atrial appendage; Ablation: AT terminated (prior acceleration)

### Classification of arrhythmias in children with TIC

Types of arrhythmias varied in the 12 children with TIC.

1. Atrial tachycardia: a total of 7 cases have been documented. Intracardiac electrophysiological mapping revealed that 57.1% of the arrhythmogenic foci were located in the right atrium, with the remaining 42.9% being left- atrial foci. Focusing on specific anatomic locations, right lung veins were the source of 28.6% of the cases, mirroring the proportion of left lung veins. The site of origin was the left atrial appendage in 14.3% of the cases and the right atrial appendage in 28.6% of the cases.
2. Atrioventricular reentrant tachycardia: Two cases of atrioventricular reentrant tachycardia were diagnosed after exclusion of atrial tachycardia. Both AVRT cases were confirmed to be associated with left-sided accessory pathways via intracardiac electrophysiological mapping, with no evidence of right-sided accessory pathways.
3. Ventricular arrhythmias: 2 cases of ventricular tachycardia and 1 case of frequent premature ventricular contractions. Among these, 2 cases of lesions were located in the left anterior fascicle and 1 case in the posterior fascicle (Table 2).

### Drug therapy and RFCA

Among the 12 children in this study, six initially received antiarrhythmic drugs such as digoxin, amiodarone, metoprolol, etc. However, the frequency and duration of tachycardia attacks in these six children were not effectively controlled by treatment. Additionally, echocardiography to assess cardiac function did not significantly improve LVEF or other relevant parameters. Due to the limited effectiveness of the drug therapy in improving arrhythmias control and improving cardiac function, all 12 children eventually received RFCA. The length of the RFCA proceedings varied considerably from one child to another. The median duration of treatment was 2 h and 9 min, with a range of 1 h and 38 min to 3 h. Two pediatric cases of atrial tachycardia showed focal points located in the thickness of the atrial cord.

The length of RFCA proceedings varied significantly between different children. The median duration of the procedure was 2 h and 9 min, with ranges ranging from 1 h to 38 min to 3 h. Two pediatric cases of atrial tachycardia showed focal points located in the atrial cord thickness. During the RFCA, atrial tachycardia was temporarily suppressed initially but recurred shortly afterward and remained sustained. As the particular anatomical structure of the atrial appendice made it difficult to perform a thorough ablation, both patients were referred to the cardiac surgical unit for the procedure. Eventually, they had an atrial appendectomy to correct the recurrent arrhythmias.

### Echocardiography and follow-up data

Echocardiography was utilized to assess left ventricular function recovery in all children during follow-up, while electrocardiographic (ECG) monitoring confirmed the absence of tachycardia recurrence in any patient. Statistical analysis of left ventricular ejection fraction (LVEF) in children who underwent radiofrequency catheter ablation (RFCA) demonstrated a significant increase in LVEF at 1 month post-RFCA compared with preoperative values (*P* = 0.002).

#### LVEF follow-up outcomes

1. At 1 month post-RFCA, 75% (*n* = 9/12) of children had an LVEF > 50%, and approximately 66.7% (*n* = 8/12) had an LVEF > 55%.
2. At 6 months postoperatively, 91.7% (*n* = 11/12) of children achieved an LVEF > 55%.
3. At 1 year postoperatively, all children (*n* = 12/12) had LVEF restored to the normal range (≥ 50%).

#### Subgroup analysis of patients with atrioventricular reentrant tachycardia (AVRT; patients 4 and 5)

1. Patient 4: Preoperative LVEF was 46%, with no prior use of antiarrhythmic drugs (AADs); LVEF remained subnormal (< 50%) until RFCA was performed.
2. Patient 5: Preoperative LVEF was 45%; short-term oral metoprolol administration only reduced tachycardia frequency, and pre-ablation LVEF remained 44% (Table 1).
3. No spontaneous normalization of LVEF was observed in either patient prior to ablation.

#### Left ventricular end-diastolic diameter (LVEDD)

In addition to LVEF recovery, most patients exhibited a clinical trend toward LVEDD normalization. However, no statistically significant differences were noted among preoperative, 1-month post-RFCA, and 6-month measurements (Greenhouse-Geisser corrected *P* = 0.333, repeated-measures analysis of variance [ANOVA]) (Table 1).

Collectively, the sustained improvement in LVEF and lack of tachycardia recurrence confirm the long-term efficacy of RFCA in restoring left ventricular function among pediatric patients with tachycardia-induced cardiomyopathy (TIC).

## Discussion

This study comprehensively reviewed clinical data from 12 pediatric patients. The results showed that in the pediatric population supraventricular tachycardia (SVT) was significantly more likely to induce TIC than ventricular tachycardia (VT). Further investigation of the distribution of SVT subtypes revealed that the primary pathogenic subtype is atrial tachycardia. It represented approximately 77.8% of all TIC cases associated with SVT, which is very similar to the findings of Hasan Candaş Kafalı [4]. Conversely, atrioventricular reentrant tachycardia represented 22.2% of TIC cases and was a subtype of the secondary pathogenic agent.

Regarding ventricular tachycardia-induced cardiovascular events, the incidence of TIC was significantly higher in patients with lesions in the branches of the heart. This finding was probably attributed to the unique electrophysiological properties of the ventricular branch region. During fascicular ventricular tachycardia, a Purkinje fibre net of branch regions had a conduction delay zone. Consequently, the impulse conduction rate was significantly lower than that of a normal Purkinje system. Moreover, the duration of refractory myocardial tissue in the reentrant circuit showed remarkable variability. These electrophysiological properties interfere with the normal cardiac electrical conduction pattern and lead to the development of a functional peripheral circuit [10, 11]. Sustained recurrent re-entrant excitation not only triggered recurrent episodes of VT, but also significantly increased the risk of fatal infections.

Patint 3 (proximal left anterior fascicle-origin VT) highlights the critical role of individualized risk assessment in VT-related tachycardiomyopathy (TIC) management. During RFCA, stimulation at the target site induced cLBBB, which, given severe preoperative left ventricular dysfunction (LVEF 24%), risked iatrogenic ventricular dysfunction. Thus, RFCA was suspended after informed consent. At 6-month follow-up, LVEF improved to 42% (from 24%), LVEDD decreased to 46 mm (from 49 mm), and tachycardia recurred. This confirms that pharmacological control plus close follow-up benefits TIC patients, supporting our “risk-benefit balancing” approach. It also guides VT management near critical conduction structures, emphasizing individualized, dynamic risk-benefit decision.

In pediatric cardiovascular disease, TIC, a specific type of cardiovascular disease, is characterised by a variety and complexity of clinical manifestations. In this study, significant differences in the clinical manifestations of TIC were observed between pediatric patients with differentiated preferences. Most of the children presented with classic symptoms such as palpitations and tachycardia. It is worth noting that three of the children did not have self-reported symptoms before the physical examination and their tachyarrhythmias were detected by chance during the electrocardiogram (ECG) examination as part of the routine physical examination. One child with TIC developed abdominal pain as a unique clinical presentation. The results of the traditional 12-lead ECG showed no obvious abnormalities, which could easily have led to misinterpretation. However, 24 h electrocardiogram monitoring has successfully detected paroxysmal atrial tachycardia, which is the cause of the disease. The child was subsequently hospitalized with a serious case of syncope. After RFCA, markers such as LVEF returned to normal, cardiac structure and function was generally normal, and the abdominal pain symptoms were completely resolved [12]. Clinicians should therefore heighten their vigilance for TIC in daily clinical practice and fully acknowledge the potential risks associated with this disease. Different evaluation methods, such as electrocardiogram monitoring and echocardiography, should be rationally selected for each pediatric condition to provide a comprehensive assessment of cardiac electrical activity, structure and function to allow early and accurate diagnosis of TIC.

Since tachycardia is a trigger for TIC, the most basic approach to treat TIC is cardiac rhythm control. Treatment options include antiarrhythmic drugs, RFCA and so on. Antiarrhythmics are not capable of eliminating the underlying mechanisms of arrhythmias [4, 8, 9]. In addition, to prevent tachycardia recurrence and avoid adverse effects of long-term Antiarrhythmic Drugs use, RFCA was performed with parental consent to target and eliminate arrhythmogenic foci. Post-operative monitoring in 12 pediatric patients showed tha RFCA was a high success in treating tachycardia of the supraventricular and ventricular tachycardia. The procedure allowed rapid restoration of left ventricular function and was associated with low complication rates. Therefore, RFCA is a safe, effective and viable treatment option for tachycardia-induced cardiomyopathy in paediatric patients.

**Fig. 1:**
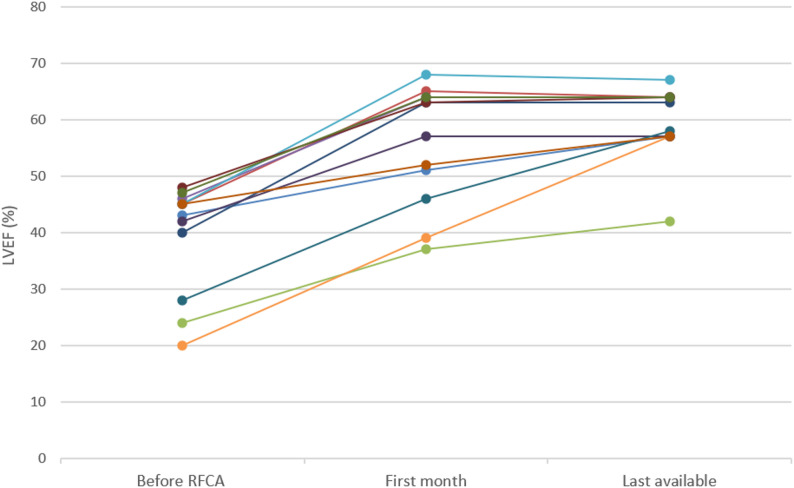
Left Ventricular Ejection Fraction (LVEF) Values Before and After Radiofrequency Catheter Ablation (RFCA)

A notable discrepancy emerged for LVEDD: while most patients (9/12) had reduced or stable 6-month LVEDD vs. baseline, repeated-measures ANOVA showed no statistical differences across preoperative, 1-month post-RFCA, and 6-month time points. This is likely due to small sample size (*n* = 12, limited power for subtle changes) and baseline LVEDD heterogeneity (30–56 mm), mildly enlarged patients (e.g., Patient 2, 35 mm) had little reduction room, while severely enlarged ones (e.g., Patient 6, preop LVEDD 56 mm) showed greater improvements, masking overall significance. Nevertheless, LVEDD improvements (especially in severe cases) remain clinically relevant, as they reflect reversed myocardial remodeling—a hallmark of true TIC (Fig. 1).

The relatively small size of the sample in our study may introduce selection bias that may affect statistical results. TIC in children usually has a favourable prognosis. However, if pediatric patients present with tachyarrhythmias and echocardiography demonstrates cardiac enlargement or dysfunction, clinicians should remain vigilant for TIC. Prompt, aggressive intervention is essential upon TIC diagnosis. Not only does early intervention improve the heart function of children, but it also reduces the occurrence of long-term cardiovascular events such as heart failure and recurrent arrhythmias. RFCA has emerged as a safe, effective treatment for pediatric tachycardia-induced cardiomyopathy (TIC), improving children’s quality of life while alleviating the financial and emotional burden on their families. To address the current study’s limitations, future research should prioritize larger cohorts. This would enable rigorous investigation of pediatric TIC diagnostic and therapeutic strategies, enhancing statistical power and thereby the reliability and generalizability of findings.

## Supplementary Information

Supplementary Material 1

## Data Availability

The datasets used and analyzed during the current study are available from the corresponding author on reasonable request.

## Abbreviations

TIC: Tachycardia-induced cardiomyopathy
RFCA: Radiofrequency catheter ablation
VT: Ventricular Tachycardia
PVCs: Premature Ventricular Contractions
AVRT: Atrioventricular Reentrant Tachycardia
AT: Atrial Tachycardia
AADs: Anti-arrhythmic Drugs
LVEDD: Left Ventricular End-Diastolic Diameter
LVEF: Left Ventricular Ejection Fraction
TNT-HS: High-Sensitivity Troponin T
CKMB: Creatine Kinase-MB
NT-proBNP: N-Terminal Pro-Brain Natriuretic Peptide
ECG: Electrocardiogram
