# Supplementary material for: Clinical significance and efficacy of radiofrequency catheter ablation in the treatment of tachycardia-induced cardiomyopathy in 12 children
Source: J Cardiothorac Surg. 2026 Jan 2;21:63. doi: 10.1186/s13019-025-03830-y (PMC12866243; doi:10.1186/s13019-025-03830-y)

**Change of authorship request form - Journals (pre-acceptance)**

**Important information. Please read.**





This form should be used by authors to request any change in authorship (adding/deleting authors) including changes in corresponding authors. This form should not be used for

name changes. Please fully complete all sections. Use black ink and block capitals and provide each author’s full name with the given name first followed by the family name.

By signing this declaration, all authors guarantee that the order of the authors are in accordance with their scientific contribution, if applicable as different conventions apply per

discipline, and that only authors have been added who made a meaningful contribution to the work.



Please note, in author collaborations where there is formal agreement for representing the collaboration, it is sufficient for the representative or legal guarantor (usually the

corresponding author) to complete and sign the Authorship Change Form on behalf of all authors, **next to the added/removed author(s)**. **(Complete Section 3, followed by Section**

**6.)**

In author collaborations where there is no formal agreement for representing the collaboration and **there are more than 10 authors**, one may sign for all, provided the signer

appends correspondence that attests that each of the authors have agreed to the change **and the added/removed authors sign the form**. **(Complete Section 3, followed by Section**

**6.)**





Please note, we cannot investigate or mediate any authorship disputes. If you are unable to obtain agreement from all authors (including those who you wish to be removed) you

must refer the matter to your institution(s) for investigation. Please inform us if you need to do this.

If you are not able to return a fully completed form within **30 days** of the date that it was sent to the author requesting the change, we may have to withdraw your manuscript. We

cannot publish manuscripts where authorship has not been agreed by all authors (including those who have been removed).





Incomplete forms will be rejected.

Please return/upload this form, fully completed, to the Journals Editorial Office. The Journal and/or Publisher will consider the information you have provided to decide whether to

approve the proposed change in authorship. We may decide to contact your institution for more information or undertake a further investigation, if appropriate, before making a

final decision.

---

Springer Nature is one of the world’s leading global research, educational and professional publishers, created in May 2015

through the combination of Nature Publishing Group, Palgrave Macmillan, Macmillan Education and Springer Science+Business Media.

---


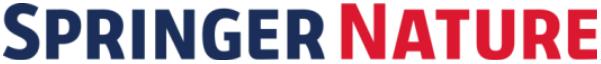

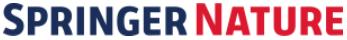

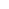


**Change of authorship request form - Journals (pre-acceptance)**

**Section 1: Please provide the current title of manuscript**

**Manuscript ID no.:** **c489bf3e-9f99-4ebf-82ea-3e976f2e30ce v2.0**

Title: Clinical Significance and Efficacy of Radiofrequency Catheter Ablation in The Treatment of Tachycardia-induced Cardiomyopathy in 12 Children

**Section 2: Please provide the previous authorship, in the order shown on the manuscript before the changes were introduced. Please indicate the corresponding author by adding (CA)**

**behind the name.**

First name(s)

Family name

ORCID or SCOPUS id, if available

1^st^ author Min Zhang

2^nd^ author Xiaoxiao Cao

3^rd^ author Yong Zhang

4^th^ author

5^th^ author

6^th^ author

th

7 author

th

8 author

th

9 author

th

10 author

Please use an additional sheet if there are more than 10 authors.

---

Springer Nature is one of the world’s leading global research, educational and professional publishers, created in May 2015

through the combination of Nature Publishing Group, Palgrave Macmillan, Macmillan Education and Springer Science+Business Media.

---


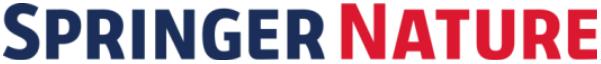

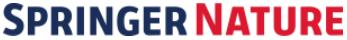


**Change of authorship request form - Journals (pre-acceptance)**

**Section 3: Please provide a justification for change. Please use this section to explain your reasons for changing the authorship of your manuscript, e.g. what necessitated the change in**

**authorship? Please refer to the (journal) policy pages for more information about authorship. Please explain why omitted authors were not originally included and/or why authors were**

**removed on the submitted manuscript.**

**T**he requested authorship change solely involves adding Xiaoxiao Cao as a co-corresponding author alongside Yong Zhang, M.D. (with Yong Zhang, M.D. listed first, then Xiaoxiao Cao); the original author list (Min Zhang, M.D., Xiaoxiao Cao, Yong Zhang, M.D.) and each author’s contribution descriptions remain unchanged.

This change is necessary because both Yong Zhang, M.D. and Xiaoxiao Cao played core roles in the study and manuscript development, fully meeting the journal’s corresponding author requirements:

- Yong Zhang, M.D. led overall study design optimization, integrated key data (organized/verified 12 pediatric patients’ baseline characteristics and post-RFCA follow-up data), validated electrophysiological findings in Table 2.docx, and oversaw academic standardization of Manuscript.docx.
- Xiaoxiao Cao collected/verified clinical data (collated preoperative/postoperative LVEDD and myocardial injury marker data in Table 1.docx), assisted in revising the manuscript’s "Results" section, and participated in formulating reviewer responses in Response to Reviewers.docx.

Xiaoxiao Cao was not originally listed as a corresponding author due to an initial oversight in designation during first submission, not insufficient contributions. All authors unanimously confirmed her contributions meet the journal’s corresponding author criteria, so this change corrects the oversight to reflect her role accurately.

No authors were removed, and the change has been unanimously approved by all authors (Min Zhang, M.D., Xiaoxiao Cao, Yong Zhang, M.D.), in line with journal policies.

**Section 4: Proposed new authorship. Please provide your new authorship list in the order you would like it to appear on the manuscript. Please indicate the corresponding author by**

**adding (CA) behind the name. If the Corresponding Author has changed, please indicate the reason under section 3.**

First name(s)

Family name (this name will appear in full on the final Affiliated institute

publication and will be searchable in various abstract

and indexing databases)

E-mail address

1^st^ author Min Zhang Wuhan Children’s Hospital  zm2005128223@163.com

2nd author Xiaoxiao Cao (CA) Wuhan Children’s Hospital  julia_4241118@163.com

3^rd^ author Yong Zhang  (CA) Wuhan Children’s Hospital 1539210298@qq.com

4^th^ author

5^th^ author

6^th^ author

7^th^ author

8^th^ author

9^th^ author

10^th^ author

Please use an additional sheet if there are more than 10 authors.

---

Springer Nature is one of the world’s leading global research, educational and professional publishers, created in May 2015

through the combination of Nature Publishing Group, Palgrave Macmillan, Macmillan Education and Springer Science+Business Media.

---


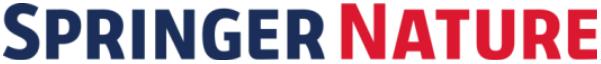

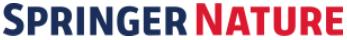

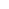


**Change of authorship request form - Journals (pre-acceptance)**

**Section 5: Author contribution, Acknowledgement and Disclosures. Please use this section to provide a new disclosure statement and, if appropriate, acknowledge any contributors who**

**have been removed as authors and ensure you state what contribution any new authors made (if applicable per the journal or book (series) policy). Please ensure these are updated in**

**your manuscript - after approval of the change(s) - as our production department will not transfer the information in this form to your manuscript.**

**New acknowledgements:** No new acknowledgements are added. Since the authorship change only involves adding a co-corresponding author (no authors removed or newly added to the author list), there is no need to acknowledge contributors who were removed as authors. No additional individuals or institutions requiring acknowledgment are mentioned in the two documents.

- **New Disclosures (financial and non-financial interests, funding):** ➀Financial Interests: All authors declare no financial relationships with any organizations that might have an interest in the submitted work in the previous three years, and no other financial interests that could be perceived as influencing the work. ➁Non-Financial Interests: All authors declare no non-financial interests (e.g., personal/professional relationships, affiliations, beliefs) that could inappropriately influence the study’s design, conduct, or reporting. ➂Funding: The study was not supported by any specific grant from public, commercial, or non-profit funding agencies (no funding information is mentioned in the two documents).
- **New Author Contributions statement (if applicable per the journal policy):**
- Min Zhang, M.D.: Participated in study design, collection of clinical data (including patient baseline information and follow-up records), and drafting of the initial manuscript.
- Xiaoxiao Cao: Responsible for clinical data verification (e.g., collating preoperative/postoperative LVEDD and myocardial injury marker data in Table 1.docx), assisting in revising the manuscript’s "Results" section, participating in formulating reviewer responses, and serving as a co-corresponding author to coordinate subsequent manuscript communication.
- Yong Zhang, M.D.: Led overall study design optimization, integrated key research data (organized/verified 12 pediatric patients’ baseline characteristics and post-RFCA follow-up data), validated electrophysiological findings in Table 2.docx, supervised the academic standardization of the entire manuscript, and served as a co-corresponding author to oversee manuscript revision and communication.

State ‘Not applicable’ if there are no new authors.

---

Springer Nature is one of the world’s leading global research, educational and professional publishers, created in May 2015

through the combination of Nature Publishing Group, Palgrave Macmillan, Macmillan Education and Springer Science+Business Media.

---


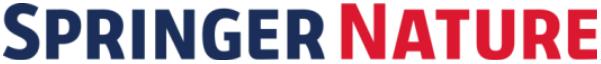

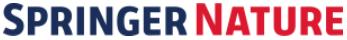

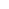


**Change of authorship request form - Journals (pre-acceptance)**

**Section 6: Declaration of agreement. *All* authors, unchanged, new and removed *must* sign this declaration.**

(NB: Please print the form, (docu)-sign and return/upload a scanned copy. Please note that signatures that have been inserted as an image file are acceptable as long as it is handwritten.

Typed names in the signature box are unacceptable.) *** Please delete as appropriate. Delete all of the bold if you were on the original authorship list and are remaining as an author.**

**
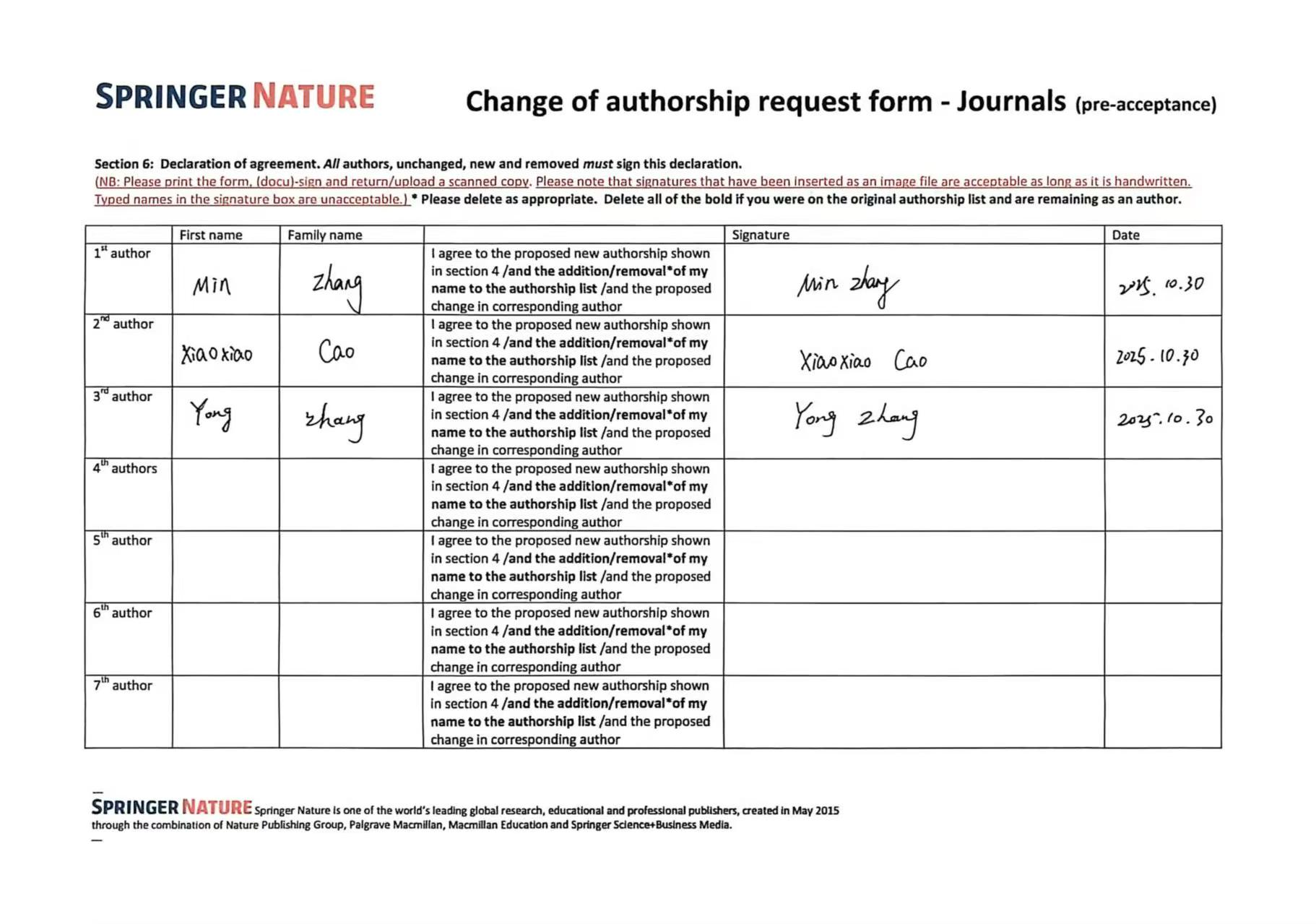
**

-

Springer Nature is one of the world’s leading global research, educational and professional publishers, created in May 2015

through the combination of Nature Publishing Group, Palgrave Macmillan, Macmillan Education and Springer Science+Business Media.

---


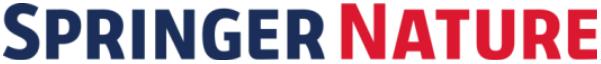

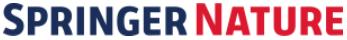

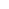


**Change of authorship request form - Journals (pre-acceptance)**

---- End of form ----

---

Springer Nature is one of the world’s leading global research, educational and professional publishers, created in May 2015

through the combination of Nature Publishing Group, Palgrave Macmillan, Macmillan Education and Springer Science+Business Media.

---


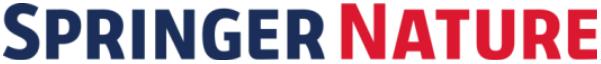

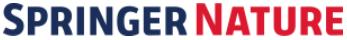

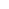

Supplement: Supplementary file 1 — Supplementary Material 1 [file 13019_2025_3830_MOESM1_ESM.docx]
